# Identification of genome-wide targets of Olig2 in the adult mouse spinal cord using ChIP-Seq

**DOI:** 10.1371/journal.pone.0186091

**Published:** 2017-10-19

**Authors:** Andrew J. Darr, Matt C. Danzi, Lee Brady, Dorothea Emig-Agius, Amber Hackett, Roozbeh Golshani, Nikita Warner, Jae Lee, Vance P. Lemmon, Pantelis Tsoulfas

**Affiliations:** 1 The Miami Project to Cure Paralysis, Miami, Florida, United States of America; 2 Department of Neurological Surgery, University of Miami Miller School of Medicine, Miami, Florida, United States of America; 3 Center for Computational Science, University of Miami Miller School of Medicine, Miami, Florida, United States of America; 4 Illumina, Inc., San Diego, California, United States of America; Instituto Cajal-CSIC, SPAIN

## Abstract

In jawed vertebrates, oligodendrocytes (OLs) are the myelin-producing glial cells responsible for ensheathment of axons within the central nervous system and are also crucial for remyelination following injury or disease. Olig2 is a crucial factor in the specification and differentiation of oligodendrocyte precursor cells (OPCs) that give rise to mature, myelin-producing OLs in the developing and postnatal CNS; however, its role in adulthood is less well understood. To investigate the role Olig2 plays in regulating gene expression in the adult OL lineage in a physiologically-relevant context, we performed chromatin immunoprecipitation followed by next generation sequencing analysis (ChIP-Seq) using whole spinal cord tissue harvested from adult mice.

We found that many of the Olig2-bound sites were associated with genes with biological processes corresponding to OL differentiation (Nkx2.2, Nkx6.2, and Sip1), myelination and ensheathment (Mbp, Cldn11, and Mobp), as well as cell cycle and cytoskeletal regulation. This suggests Olig2 continues to play a critical role in processes related to OL differentiation and myelination well into adulthood.

## Introduction

Wrapping axons in myelin sheaths has evolved in jawed vertebrates, gnatostomes, and some invertebrates as a way to increase nerve conduction velocity, enabling more rapid and efficient flow of information within the nervous system [1,2]. In the CNS, myelin is produced by OLs, making these glia cells indispensable for proper neuronal function and ultimately neural information processing. The importance of axon ensheathment is illustrated by the severe loss of function associated with demyelination following spinal cord injury or in demyelinating disorders like multiple sclerosis (MS), leukodystrophies, and peripheral demyelinating diseases [3–5]. Therefore, understanding the complex gene regulatory networks underlying myelin production and remyelination within the adult CNS is essential for developing future regenerative therapies.

During embryonic development, OPCs located within the ventricular zones (VZ) of both the brain and spinal cord produce OLs that subsequently relocate and distribute throughout the CNS. In the spinal cord, OLs are derived from both ventral, and to a lesser extent, dorsal sources within the VZ beginning around embryonic day 12.5 and 15, respectively [6–9]. Neural progenitors located in the ventral spinal cord are induced by Sonic Hedgehog (Shh) signaling, forming a discrete progenitor domain called the pMN that generates both motor neurons and OPCs in succession [10–12]. Postnatal OPCs, sometimes called polydendrocytes or NG2 cells because of the expression of the proteoglycan NG2, remain abundant in the parenchyma of the adult CNS, where they retain the ability to generate new OLs [13,14].

OL lineage progression is regulated by several stage-specific transcription factors like Olig1, Olig2, Sox10, Nkx2.2 and Nkx6.2 [15–21]. In addition, Id2, Id4, and Hes5 transcription factors must be repressed if OL differentiation is to proceed [22,23]. Furthermore, non-coding RNAs provide an additional layer of transcriptional control in development of the OL lineage. For example, miR-219 is abundant in OLs and was shown to be both necessary and sufficient for OL differentiation [24–27].

Olig2 is a basic helix–loop–helix (bHLH) transcription factor expressed at all stages of OL lineage development and is a major determination factor controlling the specification and differentiation of OPCs [17,18]. Olig2 is indispensable for the generation of motor neurons and OLs, since homozygous inactivation of Olig2 in mice results in the loss of the pMN domain and a subsequent inability to produce motor neurons and OPCs [17,18,28]. In addition, Olig2 is necessary for the development of a subset of astrocytes in both the developing (embryonic and neonatal) brain and spinal cord [29,30] and is critical for reactive astrocyte proliferation after cortical injury [31]. Olig2 mediates transcriptional regulation of effector genes through a core hexanucleotide motif CANNTG, known as an E-box, the presumptive motif recognized by bHLH factors [32].

Our current understanding of Olig2 involvement in CNS myelination and remyelination in the adult CNS remains incomplete in part because the vast majority of transcriptional targets of Olig2 have been identified using purified cells in culture or from embryonic and postnatal CNS tissues as opposed to adult tissue. Identification of genome-wide direct transcriptional targets of Olig2 in the adult CNS tissue would not only improve our understanding of Olig2 function in OPCs and mature, myelinating OLs but would help us better understand and possibly manipulate myelin production and regeneration following spinal cord injury or demyelinating diseases. To this end, we sought to map the genomic loci targeted by Olig2 in the adult mammalian CNS in vivo using ChIP-Seq from spinal cord tissue from adult mice. We subsequently chose a subset of selected ChIP-Seq targets to validate through independent qPCR experiments (ChIP-qPCR). We also identified enriched motifs, and used in-silico analyses to identify critical pathways and functional networks that were overrepresented among the genes proximal to the target sites in the ChIP-Seq dataset. Our findings provide a new resource and important insights into the gene targets of Olig2 involved in proliferation differentiation and maintenance of neuroglia, especially oligodendroglia, in the adult CNS.

## Materials and methods

### Animals

Animal usage and manipulations were performed in accordance with the University of Miami Institutional Animal Care and Use Committee and Public Health Service animal care and use guidelines.

### Chromatin immunoprecipitation (ChIP)

6-week-old male C57BL/6 mice were terminally anesthetized (ketamine/xylazine, 100/15mg, i.p.) and perfused with only cold PBS just prior to removal of the spinal cord. A total of 5 cords were aggregated for each immunoprecipitation (IP) reaction. Tissue preparation and ChIP protocol closely adhered to steps outlined in the MAGnify^™^ Chromatin Immunoprecipitation System manual (Invitrogen, 49–2024) according to manufacturer’s instructions with only a few modifications (see below). Briefly, dissected spinal cords were immediately weighed, cleaned, and minced with a razor blade in ice cold PBS. Minced tissue was then collectively homogenized by passing repeatedly through 18G and 21G needles before crosslinking chromatin with formaldehyde at a final concentration of 1% at room temperature for 10 minutes. Glycine was added to a final concentration of 0.125 M to quench formaldehyde crosslinking, and cells were then pelleted and washed with ice-cold PBS. Cell pellets were resuspended in Lysis Buffer plus Protease Inhibitors at a concentration of 1x10^6 cells / 50 μl. Nuclear lysates were then sonicated using a Branson^®^ 150 Sonifier (power setting 4, 100% duty cycle for 10 × 30-s on 90-s off intervals), yielding chromatin fragments of 50–300 bp. Dynabeads^®^ Protein A/G (Invitrogen, 10010D) were coupled to either 5 μg of Polyclonal Rabbit anti-Olig2 antibody (Millipore, A9610) or Rabbit IgG (Invitrogen, 100005291) by mixing end-over-end for 4 hours at 4°C prior to introduction to lysates. Antibody-conjugated beads were then combined with 500 μl of lysate (~1x10^7 cells) plus 500 μl of Dilution Buffer in 1.5 mL LoBind Eppendorf tubes (Sigma-Aldrich, Z666548-250EA) and mixed by end-over-end rotation overnight at 4°C. Approximately 1% of the total sheared chromatin was set aside and served as Input Control. Beads containing Protein–DNA complexes were subsequently washed with IP Buffer, formaldehyde crosslinking reversed by heat treatment, and the DNA eluted using a 12 Tube Magnetic Separation Rack (NEB, S1509S). Here, we note that the Magnify^™^ protocol is designed for use with 0.2 uL tubes, so in all subsequent steps involving end-over-end rotation we instead placed each 1.5 mL LoBind tube on its side and applied gentle agitation using a platform rocker. This small change to the protocol ensured that the beads remained submerged in solution throughout. For all steps involving bead collection via magnetic rack, 1.5 mL LoBind tubes were placed in the rack in such a way as to concentrate bead collection toward the bottom of the tube, again to keep them submerged in solution. Specifically, foam padding was added at the base of the rack to keep the tubes slightly elevated. Input Control samples were processed at the reverse crosslinking step in parallel with the ChIP’d samples. IgG IP’d DNA was analyzed by qPCR (see below) but was not included in NGS analysis.

### ChIP-Seq library preparation

DNA libraries were prepared from total sheared chromatin (Input Control Library) and ChIP-enriched fragments (ChIP’d Library) using Illumina TruSeq ChIP Library Preparation Kit (Illumina, IP-202-1024). For this experiment, we used a modified TruSeq^®^ ChIP Sample Preparation Guide protocol (Illumina, PN 15023092 Rev.B; Oct. 2013), omitting only the gel purification step to maximize total yield of adapter-ligated library fragments. Input Control and ChIP’d libraries were prepared using recommended inputs of 100 pg/μl (50 μl), for a total of 5 ng per library prep. Libraries were indexed using TruSeq Adapters AR008 (Input Control Library; ACTTGA/A) and AR001 (ChIP’d Library; ATCACG/A). All QC steps (Input DNA quantification, size distributions, and library traces) were evaluated using a Fragment Analyzer Automated CE system with High Sensitivity NGS Fragment Analysis Kit 1bp-6000bp (Advanced Analytical, DNF-474). Libraries were sequenced at 2 x 50 bp read lengths using the Illumina MiniSeq Sequencing System and MiniSeq High-Output Reagent Kits (Illumina, FC-420-1001).

### ChIP-Seq peak calling and data analysis

Sequence analysis was performed using BaseSpace Labs ChIP-Seq v.1.0.2 application (http://www.basespace.com) This application utilizes the algorithms BWA [33] to align the reads to the mouse genome, MACS2 (https://github.com/taoliu/MACS/; [34]) to identify enriched regions pulled down by ChIP, and HOMER, (http://homer.salk.edu/homer/ngs/peakMotifs.html; [35]) to identify motifs within and annotate genes proximal to these enriched regions. Custom input parameters were entered for reference genome (UCSC mouse genome; mm10) and fragment size (148 bp; Average ChIP fragment size as determined using BaseSpace BWA Aligner v1.1.4 and BAMStats 1.25; https://sourceforge.net/projects/bamstats/). Samples for both ChIP’d, and Input Control libraries were used as inputs for MACS2 to improve spatial resolution (specificity) of ChIP-region binding sites.

### ChIP-Seq quality checking

Quality checking was performed following the guidelines of the ENCODE3 Transcription Factor ChIP-Seq processing pipeline v1 (https://docs.google.com/document/d/1lG_Rd7fnYgRpSIqrIfuVlAz2dW1VaSQThzk836Db99c/). The non-redundant fraction of the aligned reads was calculated as the fraction of distinct reads (reads that align to only one genomic loci and do not have any optical duplicates) divided by the total number of reads that aligned to exactly one location. The normalized strand cross-correlation coefficient and relative strand cross-correlation coefficient were calculated using phantompeakqualtools (https://code.google.com/p/phantompeakqualtools/). These cross-correlation coefficients are relative measures of the enrichment of the ChIP data for peak-like distributions of reads (see [36] for a more complete explanation).

### ChIP-qPCR and statistical analysis

ChIP was performed using a protocol similar to the one described above for ChIP-Seq with one minor change to the fragmentation protocol- the Branson 150 Sonifier settings were changed to power setting 4, 100% duty cycle for 5 × 30-s on 90-s off intervals, yielding chromatin fragments of 100–800 bp. Primer pairs were designed using primer-BLAST (https://www.ncbi.nlm.nih.gov/tools/primer-blast/; [37]) to flank each targeted peak site. Each primer pair was subsequently cross-checked for spurious hybridization potential using UCSC In-Silico PCR web server (https://genome.ucsc.edu/cgi-bin/hgPcr?db=mm10) against both the human (hg38) and mouse (mm10) reference genomes to minimize spurious hybridization. Primers used in ChIP-qPCR are provided in Supplementary Materials. qPCR data were generated using an Applied Biosystems 7300 thermocycler and Rotor-Gene SYBR Green PCR Kit (Qiagen, 204074) according to manufacturer’s instructions. At least three independent ChIPs were generated and run in separate qPCR runs, the results are presented as means and presented in a box and whisker format using StatPlus (https://www.analystsoft.com). For between groups comparison a Student’s two-tailed t-test was used. Statistical significance was established when *p* < 0.05.

## Results

### Genome-wide mapping of direct targets of Olig2 in the spinal cords of adult mice

Expression of Olig2 in CNS oligodendroglia cells persists well into adulthood; however, it is unclear what role Olig2 transcriptional regulation plays in neuroglia, particularly OPCs and in mature OLs within the adult spinal cord, both under physiological and pathological conditions. Because of this lack of clarity, we sought to identify the genome-wide transcriptional targets of Olig2 in the adult spinal cord in vivo by performing ChIP-Seq analysis on whole spinal cord tissue harvested from healthy adult mice.

Many factors contribute to variability among ChIP-Seq datasets, so to ensure reliability of our data, and in an effort to maximize site discovery and reproducibility, we endeavored to adhere to ENCODE working standards and protocols for conducting and reporting ChIP-Seq data for point-source factors such as a transcription factor [36]. These measures included using a validated ChIP-grade antibody [38–40] and aggregating data from two independent biological experiments, resulting in approximately 55 million uniquely mapped reads, and measurement of the suggested quality control metrics. PCR duplicate analysis revealed that almost all (99.3%) of these uniquely mapped reads were non-redundant. This indicates that the sequenced DNA still retained a high degree of library complexity even after the requisite PCR steps during library preparation and, therefore, the PCR amplification likely had minimal negative impact on the results obtained. Cross-correlation analysis of the aligned ChIP-Seq data revealed evidence of successful ChIP enrichment of the type expected for a point-source transcription factor with a normalized strand cross-correlation coefficient of 1.21 and a relative strand cross-correlation coefficient of 2.98 (see Materials and methods). Each of these cross-correlation coefficients are well above the minimum levels recommended by the ENCODE consortium. Together, these quality-checking metrics support the high quality of the ChIP-Seq data presented here.

**Fig 1:**
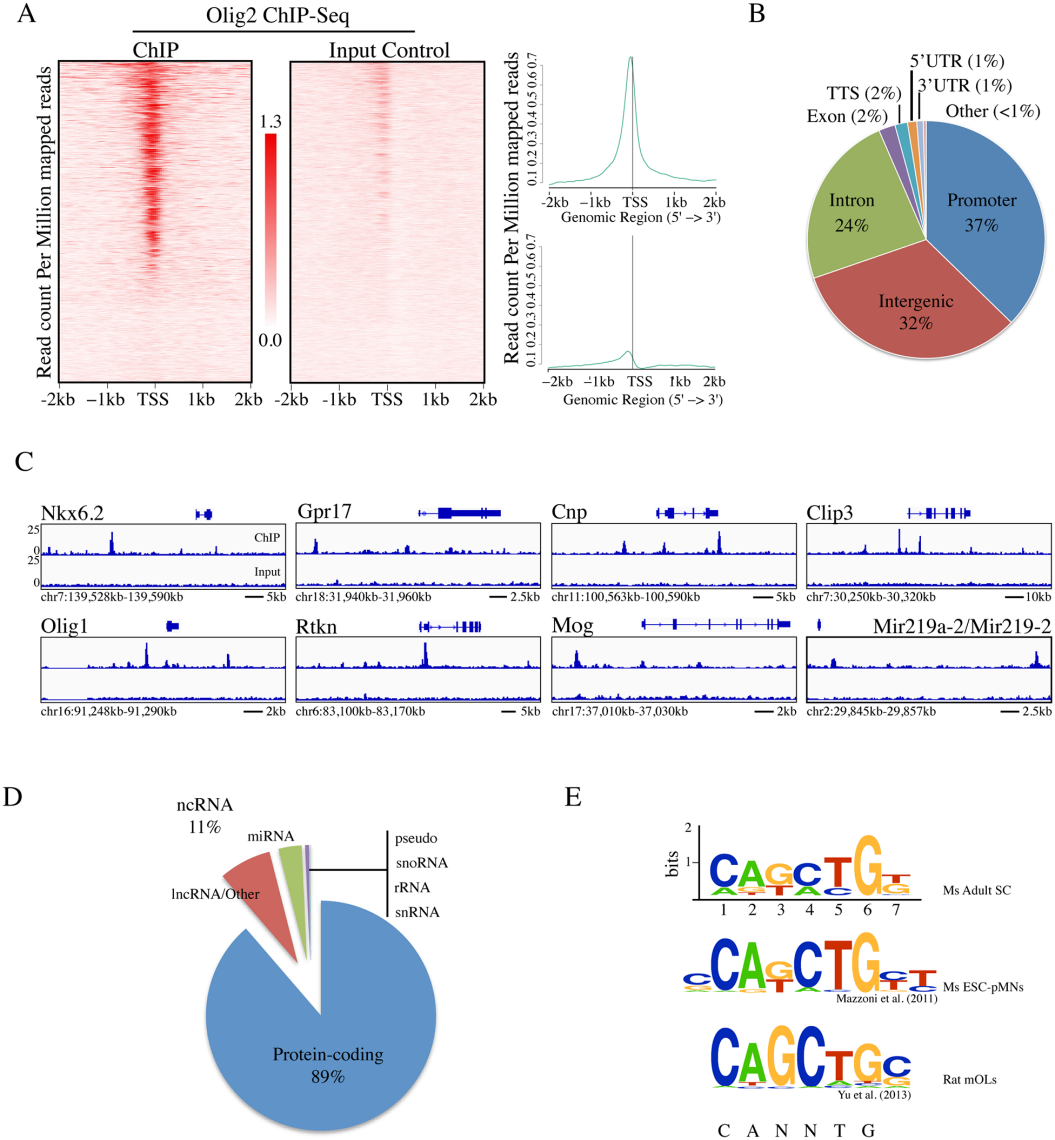
Identification of direct targets of Olig2 in the adult spinal cord in vivo. (A) Heatmap and line plot representation of ChIP-Seq signal density for Olig2 ChIP and Input Control centered on predicted TSS. (B) Pie chart showing proportions of genomic landmarks corresponding to Olig2-bound targets. (C) Integrative Genomics Viewer (IGV) visualization of Olig2 occupancy at selected target sites. (D) Pie chart showing proportions of targets with protein-coding or non-protein-coding designation. (E) De novo motif analysis of Olig2-binding regions identified a putative E-box motif (CANNTG) among ~60% of all Olig2-bound target regions. This motif configuration is consistent with previously published motif analyses for Olig2-occupancy via ChIP-Seq.

The Olig2 ChIP-Seq identified 4,214 unique regions of genomic DNA bound by Olig2 in in vivo (S1 Table; all sequences have been submitted to GEO database: GEO Series accession number GSE103324). To evaluate the global distribution of Olig2-occupied loci, we plotted the number of Olig2 sites against their distance to the nearest transcription start site (TSS). We detected a strong enrichment for Olig2 occupancy within 1 kb of the nearest annotated TSS (Fig 1A), an indication that Olig2 shows a strong occupancy bias toward target promoters. Indeed, further analysis identified 37% of peaks localized to known (annotated) promoters (Fig 1B). Comparative genomic analysis using the VistaPoint tool (http://pipeline.lbl.gov/; [41]) revealed that the majority of Olig2-occupied peaks within intergenic regions or within introns were highly conserved (S1 Fig), which suggests that they may have some functional importance. Binding regions for the transcription factor Nkx2.2 (S1A Fig) and presumptive transcription factor Zfp536 (S1B Fig) were extremely well-conserved among vertebrates, from *Homo sapiens* to *Danio rerio*. Whereas peaks of other targets like transcription factors Nkx6.2 (S1C Fig) and Klf9 (S1D Fig) were also highly conserved from *Homo sapiens* to *Gallus gallus* but were not conserved in *Danio rerio*.

To investigate whether any of the Olig2-occupied sites overlapped with genomic regions associated with known enhancer activity by comparing our ChIP-Seq peaks with the 900 enhancers that have consistent tissue-specific enhancer activity and were validated using transgenic assays [42]. Indeed, we found that one of the targeted peaks identified in the ChIP-Seq associated with the transcription factor Nkx2.2, a known target of Olig2 transcriptional regulation during development, localized to a region with demonstrated enhancer activity in vivo [42]. These findings suggest Olig2 preferentially binds to genomic regions associated with putative, and in some cases documented promoter and enhancer activity.

In our analysis, the closest annotated gene to each Olig2-binding site was considered a presumed target. Several of the targets identified in our ChIP-Seq have been identified elsewhere as direct targets of Olig2, while many genes on our list are known to be expressed in oligodendroglia, including during myelination, but were not previously known as direct targets of Olig2 (Fig 1C). For example, we identified the transcription factors Nkx2.2, Nkx6.2, and both Olig1 and Olig2, known targets of Olig2 transcriptional regulation and necessary for both OL fate specification and differentiation, as targets of Olig2 binding in the adult spinal cord in vivo (Fig 1C). We also identified Gpr17 and Zeb2/Sip1, two genes that had been reported previously as direct binding targets of Olig2 in two separate in vitro ChIP-Seq studies [43,44], as targets of Olig2 in vivo (Fig 1C). In addition to known targets, we also identified as targets of Olig2 OL-specific genes like Cnp, Mbp, Cldn11, and Sirt2, as well as myelin-associated genes like Mobp, Mog, and Mag, which were previously shown to be up-regulated 119-, 98- and 38-fold, respectively, upon OL differentiation in vitro (Fig 1C; [45–47]). Taken together, the substantial overlap between our in vivo target list and previously published lists of Olig2 targets in vitro, as well as the numerous targets we identified with OL lineage-relevant functions, strongly supports our list of in vivo targets of Olig2 generated by ChIP-Seq.

### Functional genomics of Olig2-occupied target regions

The majority of Olig2-bound sites were associated with a protein-coding gene, comprising 89%, or 3736 of the 4,214 Olig2-bound targets, while non-protein-coding RNAs made up 11% of the total target list, or 448 targets, and pseudogenes, snoRNAs, rRNAs, and snRNAs each comprised less than 1% of the total list of bound targets (Fig 1D). Of the 448 non-protein-coding targets, 137 were annotated as microRNAs (abbreviated miRNAs; Fig 1D). Notable among the list of targeted miRNAs was Mir219a-2 because it is abundant in the developing CNS, its induction coincides with OL differentiation, and it plays a critical role in facilitating the differentiation of proliferating OPCs into myelinating OLs [24–27]. Based on the results of our ChIP-Seq, we found that Olig2 occupies a region upstream of Mir219a-2 (or Mir219-2) locus and might therefore regulate Mir219a-2 in OLs in the adult spinal cord, revealing a potentially important mechanism whereby Olig2 regulates OL differentiation and myelination in the adult in vivo (Fig 1C). Unfortunately, not much known about the remaining 310 non-protein-coding targets identified in the ChIP-Seq due in part to the lack of annotation for long non-coding RNAs (lncRNAs). However, there were a few notable exceptions. For example, our data showed multiple Olig2 occupancy sites just upstream of two lncRNAs, Neat1 and Malat1/Neat2. A role for Malat1 in OL development has been suggested, however, its importance is unclear due to the lack of any clear phenotype in Malat1 knockout mice [48]. Our data suggests that Olig2 likely plays a regulatory role upstream of Malat1, Mir219a-2, and likely other non-protein-coding functional RNAs as a mechanism to control OL differentiation in the adult in vivo.

### Comprehensive de novo mapping of Olig2 targets in vivo

To investigate whether certain DNA motifs were enriched in the Olig2-binding sites, we applied a motif-discovery algorithm, HOMER [35]. Analysis revealed that the motif CA(G/T)(C/A)TG(T/G)N was statistically overrepresented (logPval = -942) among Olig2-binding sites, appearing in ~60% of them (Fig 1E). Importantly, this resolved motif contains a canonical E-box (CANNTG), the postulated target consensus of Olig2 and the bHLH family of transcription factors. We compared our motif to those previously published for Olig2, and found that our motif is nearly identical to those found in previously reported Olig2-bound targets [38,39]. In [39] for example, the authors, used the same antibody against Olig2 as the one used in this study (see Materials and methods) and reported an E box-containing motif configuration (CAGCTGC) for Olig2-occupied targets that is strikingly similar to the one identified in our de novo analysis (Fig 1E). In [38], the authors used a different Olig2 antibody to identify targets of Olig2 in mouse embryonic stem cells in vitro, yet reported a motif (CCAGCTGCT) that is very similar to ours. This notable consistency across ChIP-Seq experiments suggests that there is strong motif conservation for Olig2 occupancy across cells and developmental stages.

### Olig2 targets myelination-related genes in the adult spinal cord

**Fig 2:**
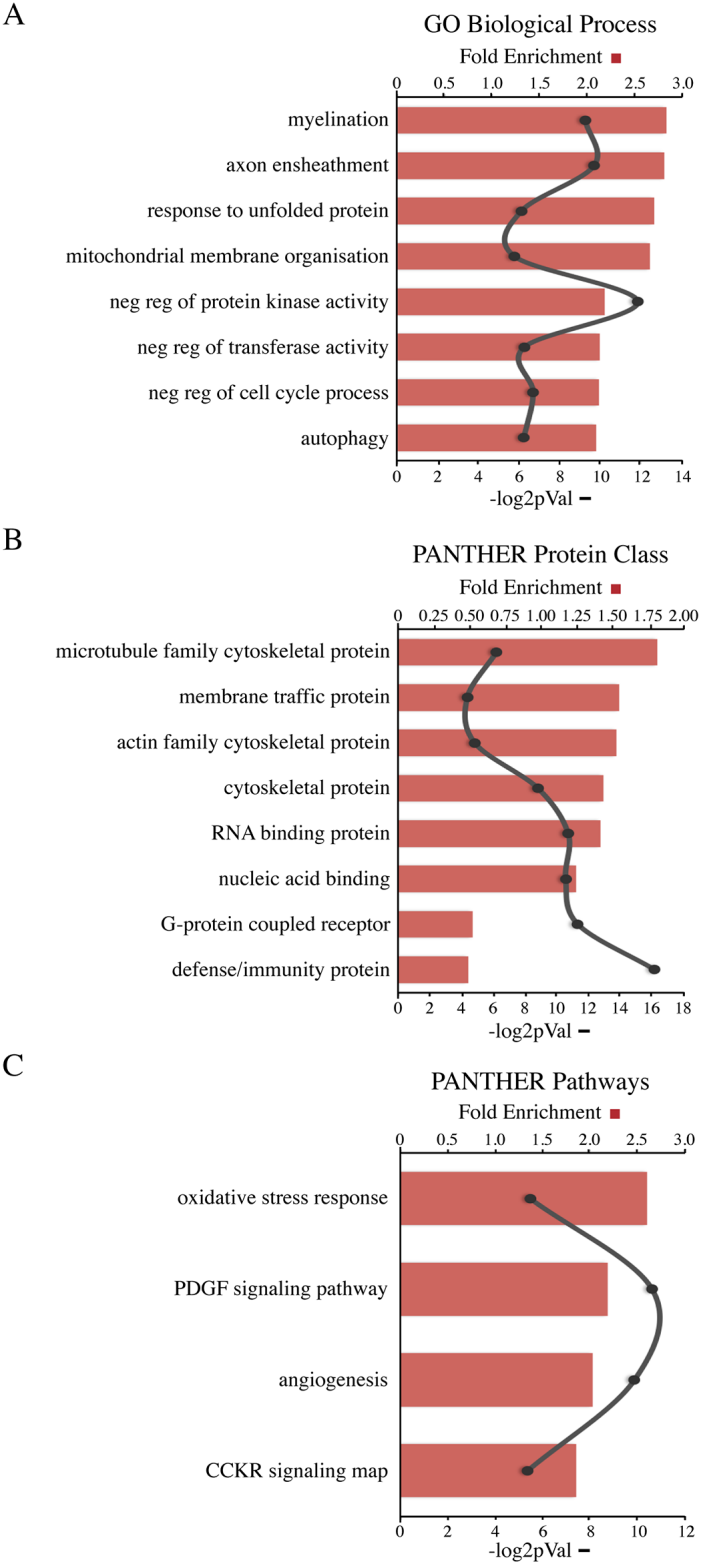
Functional GO terms overrepresented in the Olig2 ChIP-Seq dataset. Shown are the highest ranking terms for (A) Biological Processes, (B) Protein Class, and (C) Pathways according to PANTHER (geneontology.org) and ranked according enrichment score (FE; red bars) and statistical significance (-log2p-val; black line) as indicated.

To identify the major biological functions represented by the gene targets in our dataset, we looked for statistically overrepresented gene ontology (GO) terms related to biological processes using PANTHER (GeneOntology.org; [49]). The ChIP-Seq GO profile revealed a statistical overrepresentation of terms associated with oligodendroglia physiology (Fig 2 and S2 Table). The top GO terms included myelination, ensheathment, and cell cycle regulation, processes consistent with OL development (Fig 2A and S2 Table). Targets we identified with established roles in myelination and ensheathment included Mog, Mag, Mbp, Mobp, Mal, Cnp, Fth1, Opalin/Tmem10, Sirt2, and Cldn11, among many others [45–47]. We also identified Cdk4, Mcm4 and Mcm5, regulators of cell cycle/division, as targets of Olig2, suggesting that in the adult spinal cord, Olig2 continues to influence OPC proliferation via the cell cycle. In addition, we examined whether specific protein families or classes were overrepresented in our dataset. We identified an enrichment of targets associated with microtubule and actin cytoskeletal dynamics, suggesting a putative role for Olig2 in structural maintenance and remodeling of the myelin sheath (Fig 2B and S2 Table). We identified Sept9, a member of filament-forming septins that are abundant in myelin [50], and Cdc42, a member of the Rho family of GTPases required for OL maturation and formation of CNS myelin, as in vivo targets of Olig2, consistent with previous findings from a separate Olig2 ChIP-Seq study conducted in OLs in vitro [39,51]. Finally, we looked for significantly overrepresented pathways within our dataset and found that the Pdgfa signaling pathway was among the top overrepresented pathways associated with our list of ChIP-Seq targets (Fig 2C and S2 Table). Pdgf signaling pathway plays an important role in the normal development of OL lineage, as loss of Pdgfa in mice results in profound hypomyelination and reduced numbers of OPCs and OLs [52]. This further suggests that in the adult as in development, Olig2 continues to influence OPC proliferation. Taken together, the results of the GO analyses suggest that Olig2 continues to perform similar roles in adulthood as it does during late embryonic and postnatal development of the CNS, contributing to OL lineage progression and myelin production and ensheathment of axons in the adult spinal cord.

### ChIP-qPCR validation of ChIP-Seq targets

**Fig 3:**
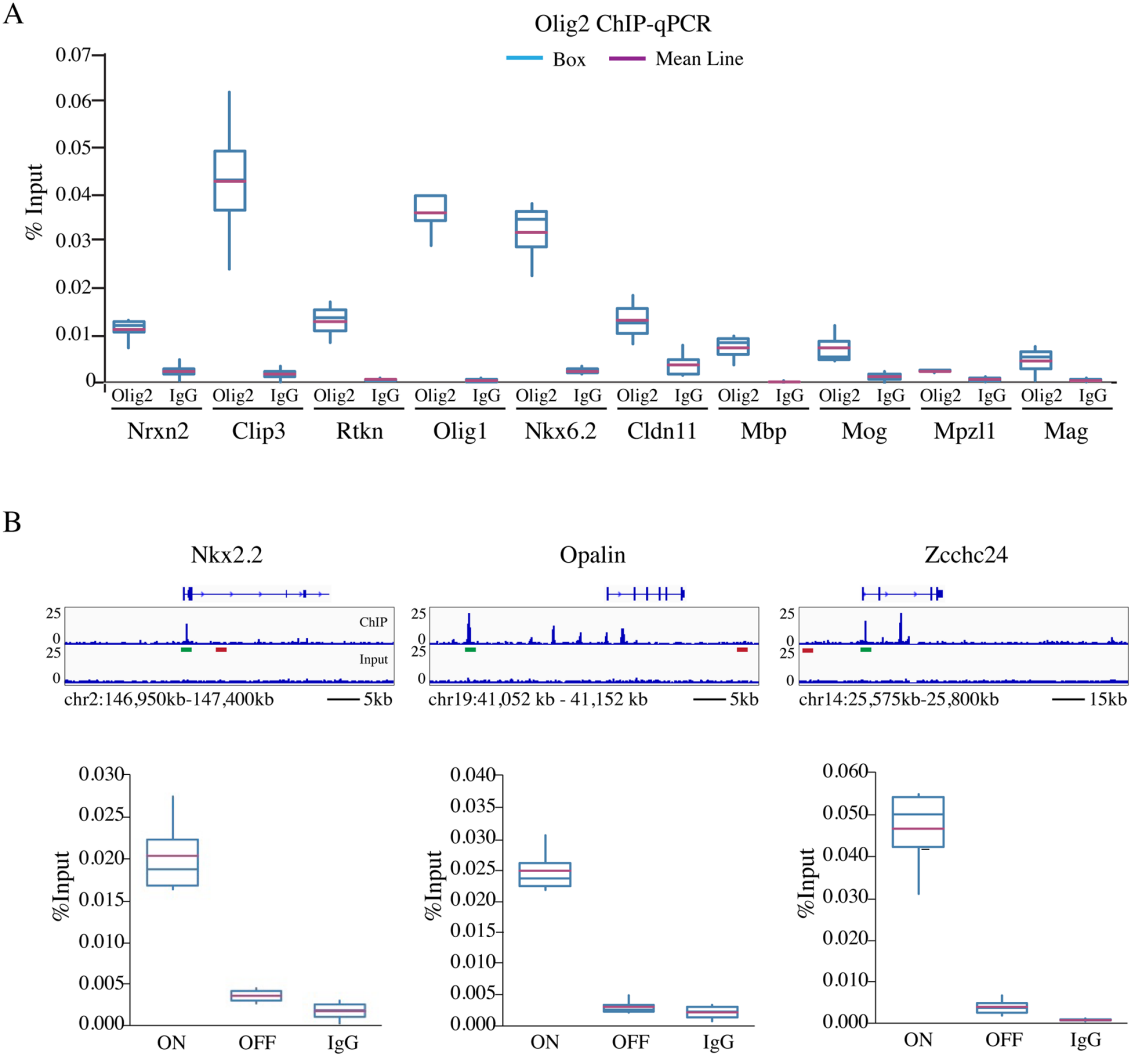
Validation of ChIP-Seq targets by ChIP-qPCR. (A) Box plot depicting enrichment over total genomic input (%Input) for both Olig2 ChIP (Olig2) and mock IgG (IgG) control conditions for 13 gene targets identified by ChIP-Seq. (B) IGV genome browser tracks showing the location of primers targeting either a specific ON-target peak summit (green bar; ON) or OFF-target region located 10–30 kb from the nearest peak (red bar; OFF) for Nkx2.2, Opalin, and Zcchc24. Results for all ChIP-qPCR data were generated from three independent experiments.

Finally, we sought to validate the ChIP-Seq results by chromatin immunoprecipitation followed by quantitative PCR (ChIP-qPCR). We included in these experiments an IgG “mock” IP condition, which served as an important negative control. For the validation, we selected 13 targets from the complete dataset, taking into account whether they had previously been identified as a direct target of Olig2 elsewhere, and whether they had a documented role in the physiology of the OL lineage. In addition, we utilized high, low, and mid-range Peak Score (PS) values, as determined by the ChIP-Seq analysis (see Materials and methods; S1 Table), when making our selection. Primers were designed to recognize the genomic region corresponding to the peak summit for each target gene, and we referred to these primer pairs as “ON-target” (S3 Table). Among the potential targets we tested by qPCR, all 13-showed enrichment as percentage of input control (Fig 3). Clip3, which had a relatively high peak score (PS = 88.28) also showed comparatively high enrichment over input (Fig 3A). Curiously, Nrxn2, which had the highest relative peak score in our subset list (PS = 94.15), and Rtkn, which also had a high peak score (PS = 74.38), showed only modest enrichment over input (Fig 3A). Previously published Olig2 targets Olig1 (PS = 62.52) and Nkx6.2 (PS = 53.86) also showed enrichment over input (Fig 3A). The other targets we tested: Cldn11 (PS = 31.26), Mag (PS = 6.70), Mbp (PS = 17.40), Mog (PS = 16.50), and Mpzl1 (PS = 10.9), showed enrichment over input commensurate with their peak scores (Fig 3A). As an additional negative control, we designed primers recognizing genomic regions 10–30 kb away from the peak summit for Nkx2.2, Opalin, and Zcchc24, which we called the “OFF-target” set of primers (Fig 3B and S3 Table). In these regions, no Olig2 peaks were detected. In contrast to the qPCR results using the ON-target primers, qPCR using OFF-target primers showed no appreciable enrichment as a percentage of input for any of the targets tested, similar to the results of the IgG mock ChIP using ON-target primers (Fig 3B). Taken together, the ChIP-qPCR enrichment data coupled with the high confidence peak summit resolution of the ChIP-Seq, demonstrate that the Olig2-bound target sites identified in the ChIP-Seq are targets of Olig2 in the adult spinal cord.

## Discussion

Despite its continued expression throughout the OL lineage well into adulthood, relatively little is known about how Olig2 functions in OL development and myelination in the mature spinal cord. In this report, we present findings from a ChIP-Seq analysis identifying direct in vivo targets of Olig2 in the adult mouse spinal cord. We found that Olig2 occupies both protein-coding and non-protein-coding targets, many with well-documented functional roles consistent with oligodendroglia physiology, in addition to numerous previously unreported targets. Moreover, de novo motif analysis revealed a binding motif that contained a canonical E-box that is strikingly similar to previously reported motifs recognized by Olig2. To our knowledge, ours is the first study to comprehensively identify genome-wide targets of Olig2 from adult mammalian spinal cord tissue.

One reason that Olig2 might remain functionally relevant in adult OLs is to activate or repress genes necessary for structural remodeling of myelin during adaptation events. The two most overrepresented GO terms for biological process were myelination and axon ensheathment while microtubule family cytoskeletal proteins were the most enriched PANTHER protein class (Fig 2A and 2B). Intracellular membrane trafficking along microtubules plays an important role in sustaining the structural and functional organization of mature OLs due to the large quantities of membrane required to form the myelin sheath, and for process outgrowth and ensheathment that involves the rapid disassembly of the actin cytoskeleton [53,54]. Our finding that cytoskeletal proteins were among the most overrepresented protein class in our dataset is consistent with previously reported results from a ChIP-Seq looking at Olig2 binding targets in mature OLs in vitro [46]. Numerous targets in our dataset have notable functional roles in either cytoskeleton dynamics related to motility/outgrowth or membrane trafficking. Sirt2, for example, is an oligodendroglia-specific protein that has been shown to inhibit OL differentiation in OPCs in vitro through deacetylation of microtubule cytoskeleton [55]. Cnp, Mbp, and Sept9, are tubulin-associated proteins that are abundant in myelinating OLs [56,57]. Clip3 is another cytoskeletal protein associated with myelin sheath formation [58]. We also identified several GTPase family members, known to be key regulators of the actin cytoskeleton. Rtkn, a downstream effector of Rho signaling, and also Cdc42, a member of the Rho family of GTPases with known roles in OL maturation and myelin sheath formation in the CNS [59,51], were both identified as targets of Olig2 in vivo. One possible role for Olig2 in differentiated OLs could be to control vesicular trafficking from the OL body to the distal myelin sheath, potentially through regulation of GTPases.

In the adult mammalian CNS, OLs continue to play crucial roles in providing both structural and metabolic support to the axons of neurons. The OLs transfer important energy metabolites, including glucose and lactate, which are important for survival of myelinated neurons, and act as anti-apoptotic and neuroprotective agents to help stave off inflammation and preserve the myelin sheath [60,61]. GO terms related to stress response and apoptotic response were also among those terms overrepresented in our dataset (Fig 2C), and there is strong evidence supporting protective roles for several identified targets in oligodendroglia in the adult CNS. For example, one of the targets identified in our ChIP-Seq was the crystallin Cryab, which is a negative regulator of inflammation with anti-apoptotic properties [62]. Mice lacking Cryab displayed worse experimental autoimmune encephalomyelitis (EAE) and inflammation than wildtype littermates, while mice treated with recombinant Cryab showed a reduction in hyper-inflammatory response and demyelination effects seen in EAE [62]. It has been proposed that another target, Gpr17 might serve as a sensor for extrinsic damage signals under pathological conditions [63]. The authors in [44], who previously showed that Gpr17 is a direct target of Olig2, demonstrated that Gpr17 is activated upon injury to OLs and that targeted inhibition of Gpr17 promotes OL remyelination, highlighting the therapeutic potential for Gpr17 in demyelinating diseases [44]. The fact that Olig2 binds to DNA in the proximity of many genes involved the production and maintenance of myelin sheaths suggests that beyond its role in cell fate determination and differentiation Olig2 might also be important in controlling several cell biological processes unique to the oligodendroglia lineage.

Along these lines, Olig2 could also function as a mediator of extrinsic cues, activating or repressing downstream effector genes of different signaling pathways. We found that the Pdgfa signaling pathway was overrepresented among our list of targets (Fig 2C). Pdgf signaling is important for the survival and proliferation of OPCs, particularly in the spinal cord where deletion of Pdgf in mice lead to a substantial reduction of OPCs and OLs and severe hypomyelination [52], and is therefore consistent with a role for Olig2 in regulating OPC proliferation and perhaps OL differentiation in the adult spinal cord. One major barrier to successful remyelination under pathological conditions is the presence of negative regulatory pathways that operate in the demyelinating environment, including BMP/Smads and Wnt/catenin signaling. We identified a number of targets that are downstream effectors of some of these signaling pathways including Tcf7l2 and Tcf4 that mediate Wnt/catenin signaling and coordinate timing of OL differentiation and myelinogenesis in the CNS [64]. We also observed Sip1, which antagonizes BMP receptor activated Smad activity while activating OL differentiation promoting factors [43]. In addition to Pdgf signaling, oxidative stress response and angiogenesis were two pathways that were also statistically overrepresented (Fig 2C). The high metabolic demands of OPCs as they differentiate into mature, myelinating OLs under different physiological conditions, requires an adequate blood supply of nutrients and oxygen. Under hypoxic conditions (oxygen tension), OPC development and white matter vascular development are tightly coupled events mediated by extrinsic canonical Wnt signaling and intrinsic HIF factors [65]. Our ChIP-Seq identified both Wnt7b, a pro-angiogenic factor and downstream effector of HIFs, as well as von Hippel Lindau (Vhl), which targets HIF factors for degradation under normal physiological conditions, as in vivo targets of Olig2 (S1 Table). Therefore, it is possible that in the adult CNS these molecules are involved in the remodeling of vessels during the generation of new OLs. These findings suggest that Olig2 might be a hub gene, coordinating divergent signaling cues that are important for the progression of OL specification and differentiation in the adult CNS.

The identification of numerous cell cycle genes as in vivo targets of Olig2 as well as the overrepresentation of GO terms related to cell cycle regulation are consistent with a role for Olig2 in OPC proliferation in the adult. Among the list of Olig2 targeted cell cycle genes included the cyclin kinase Cdk4, and mini chromosome maintenance proteins Mcm4, and Mcm5, DNA helicases that are required for licensing the DNA for successful replication. It is possible that Olig2 is maintained in adult OPCs as a means to directly regulate their cell cycle, and because the aforementioned targets regulate the early phases of cell cycle, it could act as the gatekeeper of OPCs entering the cell cycle from G0.

We identified several non-protein-coding targets of Olig2, including several miRNAs. miR-219 is one the most abundant miRNAs in mature OLs and is an essential factor in promoting OL differentiation, in part by suppressing cell cycle factors that promote continued OPC proliferation [24,25]. There are two paralogs of miR-219, miR-219-1 and -2, renamed Mir219a-1 and Mir219a-2 located on chromosomes 17 and 2 in the mouse genome, respectively. We identified Mir219a-2 as a direct target of Olig2 in vivo. It is unclear which paralog was being described in [25], however, there is evidence that strongly implicates Mir219a-2 in OL development and myelination in the CNS. For example, it was recently reported that the locus of Mir219a-2 is embedded within the intron of lncOL4, a non-protein-coding transcript that itself appears to regulate myelin-related gene expression [66]. Knockdown of lncOL4 lead to a decrease of Mir219a-2 abundance, leading the authors to speculate that the regulatory sequence controlling Mir219a-2 is also embedded within the lncOL4 locus and that some or all of the functional significance of lncOL4 in OL differentiation might be mediated through Mir219a-2 [66]. Two recent studies demonstrated the therapeutic potential of miR-219 for remyelination in animal models of demyelinating diseases [26,27]. In [26], it was shown that miR-219 accelerated the differentiation of mouse embryonic stem cells into OPCs that were in turn able to myelinate spinal neurons in vitro. Furthermore, transplantation of these miR-219-induced OPCs enhanced remyelination and cognition following cuprizone-induced demyelination [26]. In [27], it was demonstrated that small molecule mimics of miR-219 promoted remyelination in an EAE model of MS. It is possible that, in addition to Mir219a-2, any number of the miRs identified in our ChIP-Seq might have important functional roles in OL development and myelin formation and repair, however, they will need to be addressed in future studies.

Despite the aforementioned advantages of using whole CNS tissue to detect physiologically relevant binding sites of TFs, one drawback of this approach is that it makes it difficult to differentiate between different cells types. While the importance of Olig2 in establishing the OL lineage is well known, we cannot rule out that some targets identified in our ChIP-Seq derive from a smaller population of Olig2 expressing astroglia [29–31]. For example, we detected Olig2 occupancy at sites near Glast/Eaat1 and AldoC, two genes that are specifically expressed in astrocytes, while other astrocyte-centric genes like Gfap, Aqua4, Glt-1/Eaat2, and Aldh1l1 were not identified as targets of Olig2 in vivo [67,68]. We also note that there may be considerable overlap between binding sites associated with certain essential genes like housekeeping genes that might be shared across lineages. Despite these potential drawbacks, the fact that we cannot distinguish between Olig2 binding events in oligodendroglia and astroglia should not detract from the significance of these findings considering the prevailing functional role of Olig2 in the former cell lineage as opposed to the latter.

Finally, in addition to its expression in adult oligodendroglia, Olig2 is a key driver of tumor growth and proliferation [69,70], and is part of a core set of neurodevelopmental transcription factors capable of reprogramming differentiated glioma cells into stem-like tumor-propagating cells [71]. Interestingly, in [71], the authors reported the results of an Olig2 ChIP-Seq in dissociated glioblastomas where they identified a consensus motif very similar to ours (CAGCTG) using a different antibody against Olig2 than the one used in our ChIP experiments [71]. These data further strengthen the validity of our ChIP-Seq findings and also highlight the need to identify targets of Olig2 in vivo, as many of the downstream effector targets of Olig2 under normal conditions are likely involved in variety of pathophysiological events including cancer.

In summary, our findings help clarify the role of OPCs and OLs in the adult mammalian spinal cord by elucidating the gene regulatory network of a major transcriptional regulator of oligodendroglia, Olig2, in an adult in vivo context. In this report, we have provided a novel and comprehensive list of direct binding targets of Olig2 in vivo in the adult spinal cord that will provide candidates for future studies aimed at understanding how adult OPC differentiate and how OL function is maintained during both physiological and pathological processes.

## Supporting information

S1 Fig Comparative analysis of genomic regions bound by Olig2 in the adult spinal cord. Shown are conservation curves for the indicated organisms aligned to the mouse genome using the VistaPoint Alignment Tool (pipeline.lbl.gov) for target genes Nkx2.2 (A), Zfp536 (B), Nkx6.2 (C), and Klf9 (D). Below the VistaPoint curves are the tracks for Olig2 ChIP and Input regions from the corresponding genomic location generated using the IGV browser. A region was considered conserved if the conservation over that region met the default values for both the minimum conserved width (100 bp) and conservation identity (70%). Regions of high conservation are colored according to the annotation as exons (dark blue), UTRs (light blue), or non-coding (pink). Grey bar demarcates regions of strong conservation and corresponding Olig2 ChIP-Seq peak summit.
(TIF)

S1 Table Olig2 ChIP-Seq target list. A complete list of all targets identified by Olig2 ChIP-Seq.
(XLSX)

S2 Table PANTHER GO functional terms. Gene Ontology Analysis of Olig2-bound target genes using PANTHER, categorized according to biological process (A), molecular function (B), cellular component (C), protein class (D), and pathways (E).
(XLS)

S3 Table Primers used in ChIP-qPCR analysis. A complete list of ON-target (A) and OFF-target (B) primer pairs and their corresponding coordinates that were used in the ChIP-qPCR.
(XLSX)
