# Supplementary figures and images for: Identification of genome-wide targets of Olig2 in the adult mouse spinal cord using ChIP-Seq
Source: PLoS One. 2017 Oct 19;12(10):e0186091. doi: 10.1371/journal.pone.0186091 (PMC5648140; doi:10.1371/journal.pone.0186091)

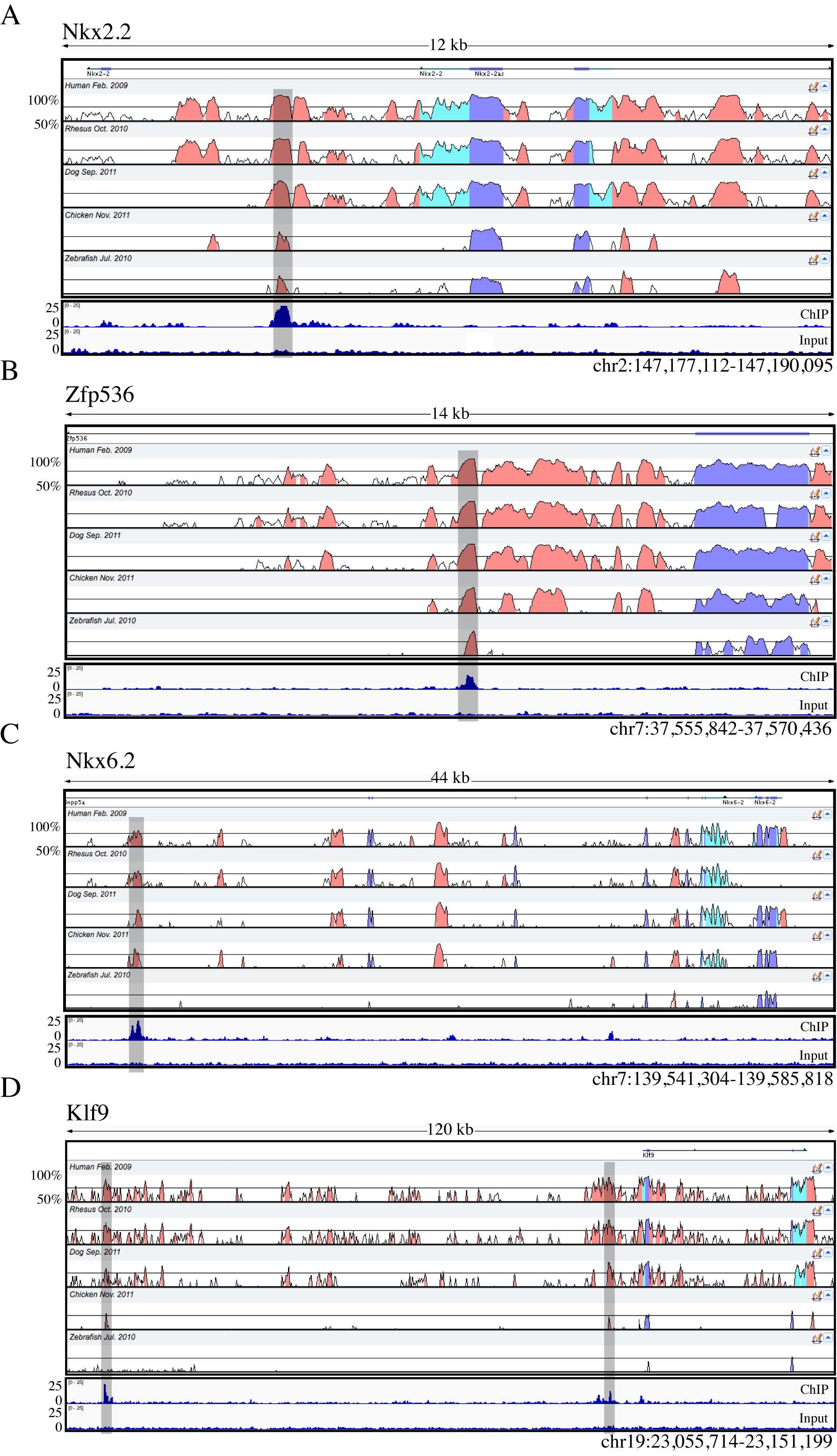

Supplement: S1 Fig — Shown are conservation curves for the indicated organisms aligned to the mouse genome using the VistaPoint Alignment Tool (pipeline.lbl.gov) for target genes Nkx2.2 (A), Zfp536 (B), Nkx6.2 (C), and Klf9 (D). Below the VistaPoint curves are the tracks for Olig2 ChIP and Input regions from the corresponding genomic location generated using the IGV browser. A region was considered conserved if the conservation over that region met the default values for both the minimum conserved width (100 bp) and conservation identity (70%). Regions of high conservation are colored according to the annotation as exons (dark blue), UTRs (light blue), or non-coding (pink). Grey bar demarcates regions of strong conservation and corresponding Olig2 ChIP-Seq peak summit. (TIF) [file pone.0186091.s001.tif]
